# Machine-Learning-Based Characterization and Inverse Design of Metamaterials

**DOI:** 10.3390/ma17143512

**Published:** 2024-07-16

**Authors:** Wei Liu, Guxin Xu, Wei Fan, Muyun Lyu, Zhaowang Xia

**Affiliations:** School of Energy and Power, Jiangsu University of Science and Technology, Zhenjiang 212003, China; liuw@just.edu.cn (W.L.); willsnx@outlook.com (G.X.); wfan@just.edu.cn (W.F.); lvmuyun125@163.com (M.L.)

**Keywords:** effective properties, Random Forest, inverse design, metamaterials

## Abstract

Metamaterials, characterized by unique structures, exhibit exceptional properties applicable across various domains. Traditional methods like experiments and finite-element methods (FEM) have been extensively utilized to characterize these properties. However, exploring an extensive range of structures using these methods for designing desired structures with excellent properties can be time-intensive. This paper formulates a machine-learning-based approach to expedite predicting effective metamaterial properties, leading to the discovery of microstructures with diverse and outstanding characteristics. The process involves constructing 2D and 3D microstructures, encompassing porous materials, solid–solid-based materials, and fluid–solid-based materials. Finite-element methods are then employed to determine the effective properties of metamaterials. Subsequently, the Random Forest (RF) algorithm is applied for training and predicting effective properties. Additionally, the Aquila Optimizer (AO) method is employed for a multiple optimization task in inverse design. The regression model generates accurate estimation with a coefficient of determination higher than 0.98, a mean absolute percentage error lower than 0.088, and a root mean square error lower than 0.03, indicating that the machine-learning-based method can accurately characterize the metamaterial properties. An optimized structure with a high Young’s modulus and low thermal conductivity is designed by AO within the first 30 iterations. This approach accelerates simulating the effective properties of metamaterials and can design microstructures with multiple excellent performances. The work offers guidance to design microstructures in various practical applications such as vibration energy absorbers.

## 1. Introduction

Metamaterials, characterized by intentionally crafted microstructures, bestow exceptional properties like a negative index of refraction, surpassing those inherent in natural materials [1]. These materials have diverse applications, including electromagnetic wave invisibility, super-resolution imaging, and acoustic lenses, making them a burgeoning focus of research marked by the creation of innovative microstructures. The pivotal role of microstructure in acquiring desired properties is evident, though its intricate arrangement poses challenges due to the complexity and interactions between constituent materials, necessitating the meticulous adjustment and characterization of effective properties by researchers [2].

Prior researchers extensively delved into this subject; for instance, Hashin and Shtrikman [3] introduced theoretical bounds for the bulk and shear modulus of two-phases and well-ordered materials using variational principles. These property bounds play a vital role in constraining effective properties and aiding in the selection of suitable phases for composite design. However, acquiring additional information about the geometric arrangement of the microstructure, a requirement for these bounds, poses difficulties [4]. To address this challenge, researchers have proposed homogenization techniques involving asymptotic expansions of governing equations to approximate material properties, such as elastic [5,6,7], thermal [5], and electromagnetic [8]. Grounded in a rigorous mathematical theory [9], this approach provides a viable solution for certain material design problems. Numerical techniques like finite-element analysis (FEM), boundary element methods, or spectral methods can be employed to solve homogenization equations, with FEM standing out as a popular choice. Numerous investigations have been conducted to investigate the properties of composite materials, employing a combination of experimental techniques and FEM simulations. B. Panda et al. [10] utilize experimental techniques to test the properties of geopolymer. X. Ren et al. [11] study the mechanical properties of foam-filled auxetic circular tubes with experiments and FEM simulations. B Yuan et al. [12] investigate the impact resistance and interaction mechanism of glass fiber using drop-weight tests. Despite their utility, traditional FEM often faces challenges in terms of computational cost to characterize the properties of metamaterials. Hence, an urgent need exists for an innovative approach to expedite the discovery and design process for novel materials. The rapid advancements in computer science have fortunately paved the way for researchers to harness the potential of machine-learning approaches. Machine learning offers advantages such as low computational cost, short development cycles, robust data processing capabilities, and high predictive performance [13]. Its applications have permeated various domains, such as medical [14], bioinformatics [15], and acoustic [16], with a notable focus on materials discovery and structure design, yielding significant improvements in both time efficiency and prediction accuracy. Diverse machine-learning models have been proposed to predict various physical properties and behavior. For instance, Na et al. [17] employ graph neural networks to identify structures and make precise predictions regarding the band gap of crystalline compounds, a crucial acoustical property. Similarly, A.Y. Churyumov et al. [18] use ANN (Artificial Neural Networks) to predict the bending behavior of line heating metal, and R. Honysz [19] utilizes ANN, MLP (Multilayer Perceptron) and GRNN (Generalized Regression Neural Networks) to predict chemical composition of alloying elements in steels. While existing research has predominantly concentrated on predicting physical and chemical properties using machine learning, there remains limited exploration into obtaining effective properties by homogenizing metamaterials and optimizing structures on the base of predicted properties.

This paper introduces a machine-learning-based approach for predicting effective properties and designing microstructures with exceptional characteristics. To facilitate the machine-learning process, various structures with diverse geometrical parameters are constructed, and numerical homogenization is applied to determine the effective properties of metamaterials. Using these results, a Random Forest (RF) [20] machine-learning model is established to learn from the data and predict effective properties. The paper also addresses optimization and structural design challenges, employing the Aquila Optimization (AO) [21] algorithm and comparing its performance with two other algorithms to determine the optimal parameter combination for inverse design. Results indicate that the algorithm can identify suitable microstructure parameters, albeit with a slightly longer computational time. This method underscores the capability of machine-learning algorithms to expedite metamaterial characterization, accelerating the metamaterials research. Moreover, a multi-objective optimization problem is solved to design a structure with multiple excellent performances.

The subsequent sections of this paper are structured as follows: In Section 2, we provide an introduction to numerical homogenization theory, the RF algorithm, and the AO algorithm, elucidating their roles in calculating effective properties, model training, and inverse design, respectively. Section 3 outlines a scheme for prediction and inverse design. The outcomes of prediction and inverse design are detailed in Section 4. Finally, Section 5 presents the conclusions drawn from this study.

## 2. Methods

### 2.1. Numerical Homogenization Theory

**Figure 1:**
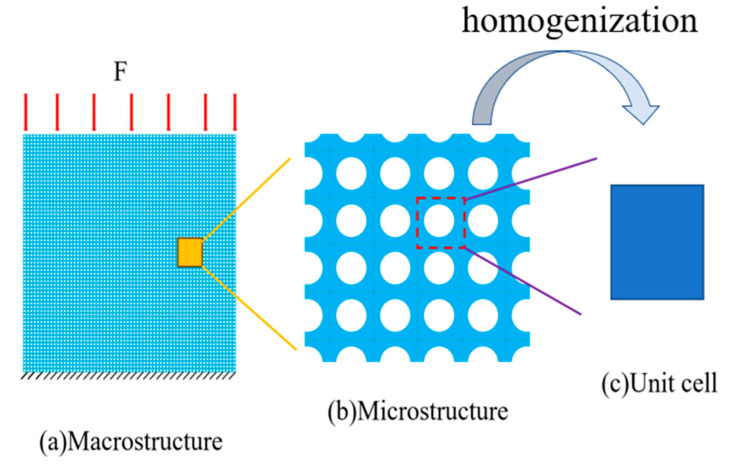
Homogenization process (**a**) Macrostructure, (**b**) Microstructure, and (**c**) homogenized unit cell.

Numerical homogenization theory is a widely employed methodology for tackling homogenization equations. As depicted in Figure 1, the diagram illustrates the macro, micro, and homogenized unit cells of a periodic structure. A key concept in numerical homogenization is the Representative Volume Element (RVE), as shown in Figure 1c. In this study, we initially employ the finite-element method (FEM) to compute the effective properties of metamaterials and generate the necessary training data. The assumption within the FEM model is that the constitutive law follows linear elasticity, and it is further hypothesized that the size of the periodic RVE is significantly smaller than the size of the macrostructure depicted in Figure 1a. Given the abundance of literature introducing FEM for numerical homogenization to numerically determine the effective medium parameters [22,23], a brief introduction is presented here. The displacement under the macroscopic scale is $x_{i}$, while the displacement under the microscopic scale is denoted as $y_{i}$. The relationship between the local coordinate system $y_{i}$ for the RVE and global coordinate system for macrostructure $x_{i}$ can be defined as

(1) $$y_{i}=\frac{x_{i}}{\epsilon }$$

where ϵ is a very small scaling parameter between these two length scales.

The asymptotic expansion form of macroscopic displacement can be written as:

(2) $$u(x)={u_{i}}^{0}(x,y)+\epsilon^{1}{u_{i}}^{1}(x,y)+\epsilon^{2}{u_{i}}^{2}(x,y)+\cdots$$

where $x=(x_{1},x_{2},x_{3})$ and $y=(y_{1},y_{2},y_{3})$, u refers to displacement.

By utilizing periodic boundary conditions, the characteristic equations are solved, and the expression for the homogenized elastic constant can be obtained:

(3) $$D_{ijkl}^{H}\left(x\right)=\frac{1}{\left|Y\right|}\int_{Y}\left[E_{ijkl}-E_{ijpq}\frac{\partial X_{p}^{kl}}{\partial y_{q}}\right]dY$$

where $D_{ijkl}^{H}$ is homogenized elastic constant, $X_{p}^{kl}$ is a characteristic function obtained by solving non-homogeneous integral equations, $E_{ijkl}$ refers to elastic tensor, $\left|Y\right|$ refers to the volume of the unit cell.

### 2.2. Random Forest (RF) Algorithm

The RF algorithm, initially proposed by Breiman [20], is an ensemble learning method applicable to both classification and regression tasks. In the context of regression, RF is composed of decision trees $\left\{h\left(x,\theta_{k}\right),\;k=1,\cdots ,n\right\}$. The prediction result of RF is obtained as an unweighted average of all the prediction results from regression trees:

$$\bar{h}\left(x\right)=\frac{1}{n}\sum_{1}^{n}h(x,\theta_{k})$$

where x is the input vector, n is the number of decision trees, $\theta_{k}$ is an identically distributed random vector, x and $\theta_{k}$ are independent of each other.

The mean-squared error for any predictor h(x) can be expressed as

$$error={E_{x,y}(y-h(x))}^{2}$$

where y is a real value.

**Figure 2:**
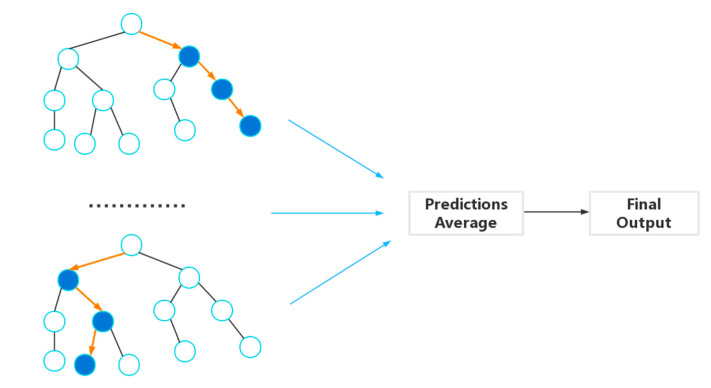
The diagram of the RF method.

The structure of RF has made noticeable improvements in the accuracy of learning by classification/regression and falls within the sphere of ensemble learning [24]. Figure 2 shows a diagram of the RF method.

### 2.3. Aquila Optimizer

Aquila Optimizer (AO) is a novel meta-heuristic optimization algorithm. In AO, the optimization is based on a set of solutions (X) shown in Equation (4). These solutions are randomly generated as initial solutions between the upper bound (MAX) and lower bound (MIN) of the problem, which ought to be optimized.

(4) $$X=\left[\begin{array}{ccc} X_{1,1} & \cdots & X_{1,n}\\ \vdots & \ddots & \vdots \\ X_{N,1} & \cdots & X_{N,n} \end{array}\right]$$

where X refers to candidate solutions, N specifies the number of the solutions in X, n is the dimension size of the test problem.

$X_{i}$ is considered as the strategy of ith solution, N narrates the number of exploited candidate solutions, and n refers to the number of used locations in the test problem.

(5) $$\begin{array}{c} X_{ij}=rand\times ({MAX}_{j}-{MIN}_{j})+{MIN}_{j}\\ i=1,2,\cdots ,N\\ j=1,2,\cdots ,n \end{array}$$

where rand is a random number.

Four hunting methods are employed selectively by Aquila. The mathematical description of the AO process is given as follows:

(1) The first hunting strategy ($X_{1}$), Aquila searches the prey extensively at a high level. The strategy is defined as follows:

(6) $$X_{1}(t+1)=X_{best}(t)\times (1-\frac{t}{T})+(X_{M}(t)-X_{best}(t)*rand)$$

where $X_{best}(t)$ refers to the best solution, ($1-\frac{t}{T}$) is used to control the process of iteration, $X_{M}(t)$ is defined by Equation (7) with the purpose of finding the local mean value of the current solutions.

(7) $$X_{M}\left(t\right)=\frac{1}{N}\sum_{i=1}^{N}X_{i}\left(t\right),\;j=1,\;2,\;\cdots ,\;n$$

(2) The second hunting strategy ($X_{2}$), Aquila finds the target and circles above. This behavior can be mathematically presented as:

(8) $$X_{2}(t+1)=X_{best}(t)\times Levy(D)+X_{R}(t)+(y-x)\;*\;rand$$

where Levy(D) is the levy flight distribution function, and $X_{R}(t)$ is a selected random solution.

(9) $$Levy(D)=s\times \frac{u\times \sigma }{{\left|v\right|}^{\frac{1}{\beta }}}$$

where s is fixed to 0.01, u and v are random values, and σ is defined by Equation (10):

(10) $$\sigma =\left(\frac{\Gamma \;(1+\beta )\times sin(\frac{\pi \beta }{2})}{\Gamma (\frac{1+\beta }{2})\times \beta \times 2^{\left(\frac{\beta -1}{2}\right)}}\right)$$

where β is fixed to 1.5.

In Equation (8), *y* and *x* are employed to indicate the spiral form:

(11) y=r×cos⁡(θ)

(12) x=r×sin⁡(θ)

where

(13) $$r=r_{1}+U\times D_{1}$$

(14) $$\theta =-\omega \times D_{1}+\theta_{1}$$

(15) $$\theta_{1}=\frac{3\times \pi }{2}$$

$r_{1}$ ranges in $\left[\begin{array}{cc} 1 & 20 \end{array}\right]$, U is settled as 0.00565, $D_{1}$ ranges in $\left[\begin{array}{cc} 1 & n \end{array}\right]$, and ω is fixed at 0.005. All parameter values are taken from the original paper [21].

(3) The third hunting strategy ($X_{3}$), Aquila prepares to attack around the area of prey. Strategy can be presented as:

(16) $$\begin{array}{c} X_{3}(t+1)=(X_{best}(t)-X_{M}(t))\times \alpha -rand\\ +((MAX-MIN)\times rand+MIN)\times \beta \end{array}$$

where α and β are parameters set as constant 0.1.

(4) The fourth hunting strategy ($X_{4}$), Aquila grabs the prey. The behavior can be mathematically presented as given in Equation (17):

(17) $$\begin{array}{c} X_{4}(t+1)=QF\times X_{best}(t)-(G_{1}\times X(t)\times rand)\\ -G_{2}\times Levy(D)+rand\times G_{1} \end{array}$$

where QF denotes a quality function used to promote the search process, $G_{1}$ is generated by Equation (19), $G_{2}$ is generated by Equation (20), and X(t) refers to the current solution.

(18) $$QF(t)=t^{\frac{2\times rand-1}{{\left(1-T\right)}^{2}}}$$

(19) $$G_{1}=2\times rand-1$$

(20) $$G_{2}=2\times (1-\frac{t}{T})$$

## 3. RF-Based Computational Scheme for Effective Properties and Inverse Design

**Figure 3:**
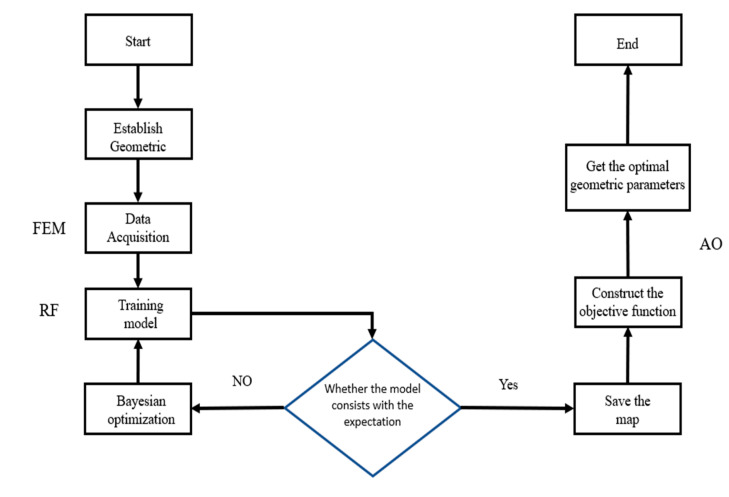
The diagram of the proposed framework.

In this section, we outline the development of the RF-based computational scheme for effective properties and inverse design. The construction process details are introduced, and the comprehensive workflow is illustrated in Figure 3.

### 3.1. Representative Topology Construction

First, we provide a brief overview of the development of the 2D and 3D unit cells of metamaterials.

2D: Parametric curves

$$\left[\begin{array}{c} x^{\prime }\\ y^{\prime } \end{array}\right]=\left[\begin{array}{cc} \cos b & -\sin b\\ \sin b & \cos b \end{array}\right]{\left[\left|\cos\frac{m\times t}{4}\right|+\left|\sin\frac{m\times t}{4}\right|\right]}^{n}\times \left[\begin{array}{c} ss\cos t\\ \sin t \end{array}\right]$$

$$\left[\begin{array}{c} x\\ y \end{array}\right]=\left[\begin{array}{c} x^{\prime }\\ y^{\prime } \end{array}\right]\sqrt{\left(1-pr)/(ss\;A_{0}\right)}$$

where m means rotating geometric features 360°/m without changing, ss is the scale factor, t∈(−π,π), b controls the rotation, $A_{0}$ is the area of the geometric, and pr is the ratio of the scatter area to the whole area.

3D: Triply periodic minimal surface

Triply periodic minimal surfaces (TPMS) [25] are mathematically defined structures that repeat in three dimensions with zero mean curvatures and large surface areas. Two of the classic structures are the Schwarz P surface proposed by Schwarz in 1865 and the Gyroid surface proposed by Luzzati in 1967. These surfaces are described by the following approximate equations:
Schwarz P:U=cos⁡(2πx)+cos⁡(2πy)+cos⁡(2πz)Gyroid:U=cos⁡(2πx)sin⁡(2πy)+cos⁡(2πy)sin⁡(2πz)+cos⁡(2πz)sin⁡(2πx)

In this paper, a form of transformation is applied to the function of TPMS:
Roll:$R_{\varphi }=\left[\begin{array}{ccc} \cos\varphi & 0 & -\sin\varphi \\ 0 & 1 & 0\\ \sin\varphi & 0 & \cos\varphi \end{array}\right]$
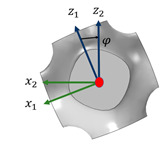
Pitch:$R_{\theta }=\left[\begin{array}{ccc} 1 & 0 & 0\\ 0 & \cos\theta & -\sin\theta \\ 0 & \sin\theta & \cos\theta \end{array}\right]$
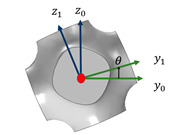
where φ and θ refer to rotation angle based on Y-*axis* and X-*axis*, respectively.

The final formulation of transformed TPMS is
f ($x^{\prime }$,$y^{\prime }$,$z^{\prime }$):$\left(\begin{array}{c} x^{\prime }\\ y^{\prime }\\ z^{\prime } \end{array}\right)=R_{\varphi }R_{\theta }\left(\begin{array}{c} x\\ y\\ x \end{array}\right)$

**Figure 4:**
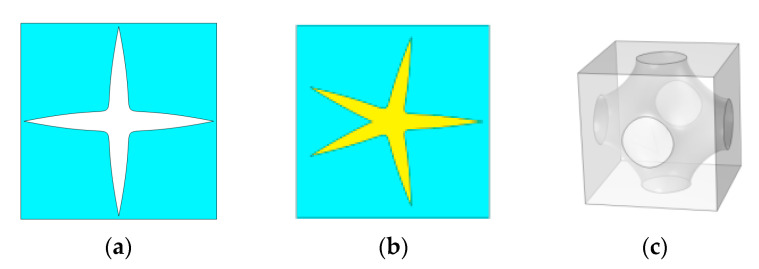
Three representative constructions of metamaterials. (**a**) Porous metamaterials. (**b**) 2D metamaterials with solid–solid-based materials. (**c**) 3D metamaterials with fluid–solid-based materials.

Three representative constructions of metamaterials are shown in Figure 4. Many graphical parameters are considered, and the detailed parameter settings are described in Section 4.

### 3.2. Data Acquisition

Following the unit cell construction, numerical homogenization using COMSOL 6.0 and ABAQUS 2018 is applied to compute the effective thermal conductivity, effective Young’s modulus, and effective Poisson’s ratio. For 2D, 7560 triangle elements are built, and periodic boundary conditions are applied to the four edges in COMSOL and ABAQUS. For 3D, 108898 tetrahedral elements are built, and the boundary layer is finely meshed. To assemble a comprehensive dataset for model training, various parameters are systematically adjusted to modify the geometry. For enhanced accuracy and a thorough mapping of performance between structures and effective properties, parameter “m” is varied from 1 to 10, parameter “ss” is varied from 1 to 2.5, and “n” is varied from 0 to 8. A total of 1030 sets of data are generated through FEM simulations.

### 3.3. Training and Prediction Process of RF

Using the acquired datasets, RF constructed in MATLAB is employed to predict the properties of metamaterial. Data processing commonly involves two methods: standardization and normalization, and in this work, normalization is applied. In the training process of RF, Bayesian optimization is utilized to fine-tune the hyperparameters, with the goal of minimizing the out-of-bag error. These hyperparameters encompass samples of one leaf node, the number of estimators, and the maximum depth of the tree. To assess the model’s generalization ability and accuracy, three indicators—root mean square error (RMSE), mean absolute percentage error (MAPE), and $R^{2}$, are employed to evaluate the RF model’s performance. RMSE measures the deviation between predictions and true values, MAPE calculates the average percentage of absolute error between predictions and true values, and $R^{2}$ gauges the accuracy of the predicted results. The mathematical forms are defined as follows:

$$RMSE={\sqrt{\frac{1}{n}\sum_{i=1}^{n}\left(y_{i}-\hat{y_{i}}\right)}}^{2}$$

$$MAPE=\frac{100\%}{n}\sum_{i=1}^{n}\left|\frac{y_{i}-\hat{y_{i}}}{y_{i}}\right|$$

$$R^{2}=1-\frac{\sum_{i}{\left(\hat{y_{i}}-y_{i}\right)}^{2}}{\sum_{i}{\left(\bar{y_{i}}-y_{i}\right)}^{2}}$$

where $\hat{y_{i}}$ refers to the value predicted by the model, $\bar{y_{i}}$ is the average value of true values, $y_{i}$ represents true value, and n means the number of the data.

### 3.4. Inverse Design with AO

In addition to predicting the properties of metamaterials using RF, inverse design is a crucial aspect of metamaterial research. In general, inverse design can be framed as an optimization problem. Specifically, the optimization problem addressed in this paper aims to minimize the effective thermal conductivity while ensuring a large value for the effective Young’s modulus. The exploration of an optimal parameter combination, namely “m” and “ss”, is undertaken, where “m” ranges from 4.05 to 6, and “ss” varies from 1 to 1.1. The general mathematical formulation of multi-objective optimization can be expressed as follows:

$$X=\left[\begin{array}{ccc} x_{1,1} & \cdots & x_{1,N}\\ \vdots & \ddots & \vdots \\ x_{N,1} & \cdots & x_{N,N} \end{array}\right]$$

$$Y=[y_{1},y_{2},\cdots ,y_{n}]=Mdl(X)$$

$$optimization\;function(o)=w_{1}*\frac{{Mdl}^{1}(X)}{Max(Y_{1})}+w_{2}*\frac{{Mdl}^{2}(X)}{Max(Y_{2})}+\cdots$$

where $x_{i,j}$ is the variable of the geometry parameters. Y is the value of effective material properties of different parameters. The optimization function is utilized to describe the test problem in a mathematical form. Mdl is a model constructed by RF to establish the relation between X and Y. $w_{i}$ denotes a weight used to set the preference when the objects are in conflict.

## 4. Results and Discussion

### 4.1. The Effective Properties of 2D Metamaterials

In this section, we delve into the influence of geometrical parameters on two-dimensional structures. All reference results are acquired through FEM simulations with a highly refined mesh, ensuring precision and accuracy in the analysis. The Young’s modulus of the matrix is 108 (GPa), Poisson’s ratio of the matrix is 0.3, and the thermal conductivity of the matrix is 10 (W/mK). The real material of the matrix is Ti. The Poisson’s ratio of the scatter is 0.45, and the thermal conductivity of the scatterer is 60 (W/mK). The real material of the scatterer can be a kind of metal alloy.

#### 4.1.1. Effective Material Properties of 2D Porous Metamaterials

**Figure 5:**
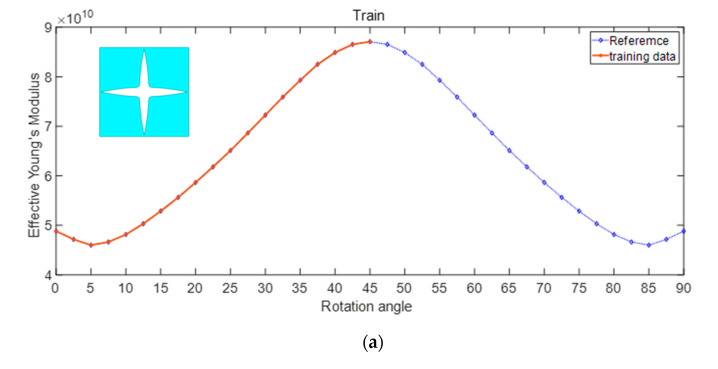

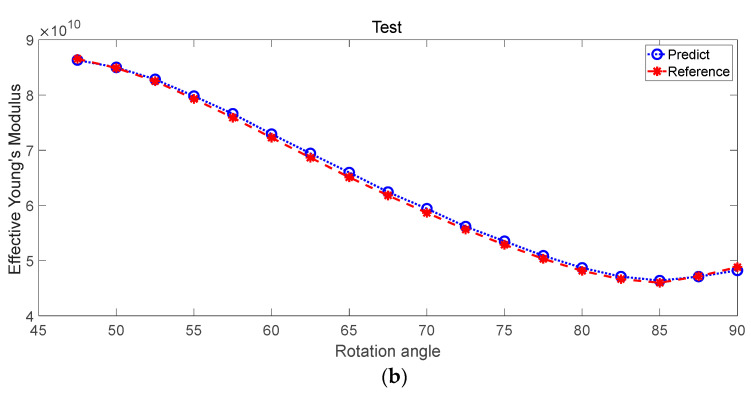
Influence of rotation angle on effective Young’s modulus (**a**) proportion of training set (**b**) prediction.

Initially, we investigate the impact of the rotation angle on the effective Young’s modulus and Poisson’s ratio of porous metamaterials. The geometric parameters are set to m = 4, n = −5, pr = 0.88, $A_{0}$ = 0.512, and the lattice constant is a = 10 (mm). The effect of the rotation angle on the effective Young’s modulus is illustrated in Figure 5. In Figure 5a, only 50% of data are utilized as training data. It is noticed that the geometric is symmetrical. The effective Young’s modulus will also exhibit symmetry with changes in rotation angle. The regression model can learn the trend according to the features, and the prediction results are very accurate, as shown in Figure 5b.

**Figure 6:**
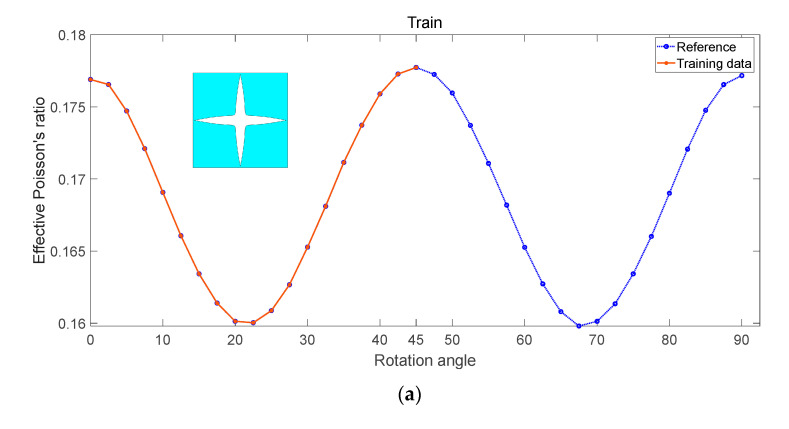

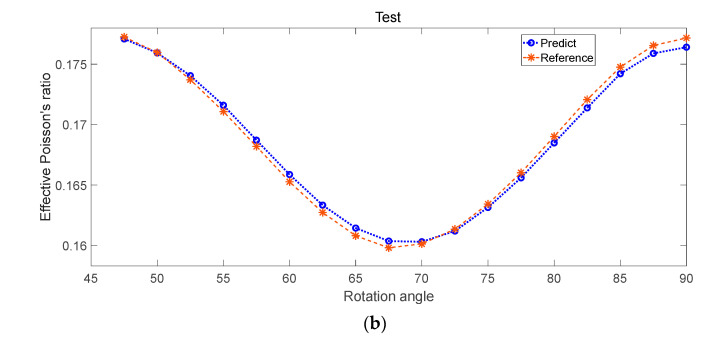
Influence of rotation angle on effective Poisson’s ratio (**a**) proportion of training set (**b**) prediction.

Young’s modulus is an indicator of a material’s hardness, whereas Poisson’s ratio characterizes the extent of transverse and longitudinal deformation when subjected to a load. Although both are vital material properties, Poisson’s ratio is often overlooked. This study utilizes a consistent porous structure to investigate how geometric parameters affect the effective Poisson’s ratio. In Figure 6a, half of the data are used as training data, and the prediction outcomes, utilizing the RF model, are presented in Figure 6b. The symmetry of the graph still plays a crucial role in predicting Poisson’s ratio. Interestingly, the model thinks Poisson’s ratio is still symmetrical, but there is a point deviation compared to the true value. Generally, these predictions align closely with the reference results, underscoring the model’s accuracy.

#### 4.1.2. Effective Material Properties of 2D Solid–Solid Metamaterials

**Figure 7:**
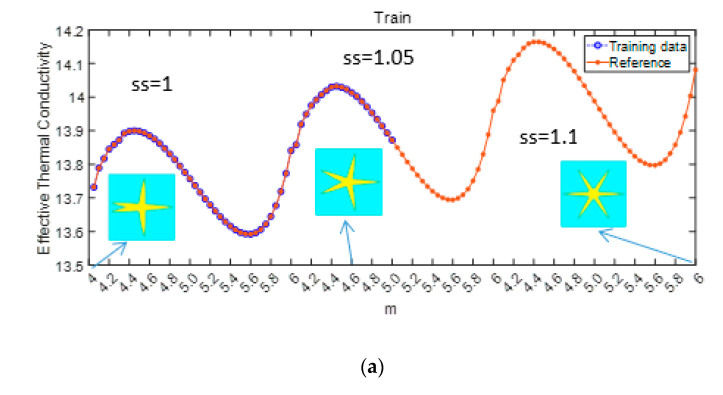

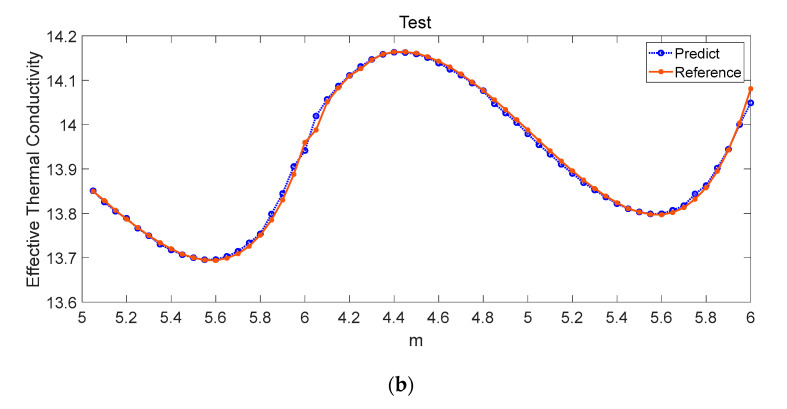
Influence of graph parameters m and ss on effective thermal conductivity (**a**) proportion of training set (**b**) prediction.

In this section, the investigation expands to include solid–solid-based materials and associated thermal properties are taken into consideration. The initial structure of the metamaterial is depicted in Figure 4b. To introduce complexity into the dataset, the impact of two variables on the effective properties is explored. The influence of geometric parameters “m” and “ss” on effective thermal conductivity is illustrated in Figure 7, where “m” ranges from 4.05 to 6, and “ss” varies from 1 to 1.1. As shown in Figure 7a, the effective thermal conductivity exhibits periodicity due to the influence of the geometric parameter “m”, where “m” represents the rotation of geometric features by 360°/m without changing—a primary reason for the observed periodicity. Notably, the overall trend of effective thermal conductivity shows a gradual increase with the influence of the factor “ss”. Following the same methodology as the porous material, 50% of the data are used as the training set. Figure 7b demonstrates that the RF model successfully captures the trend of the data and makes accurate predictions.

**Figure 8:**
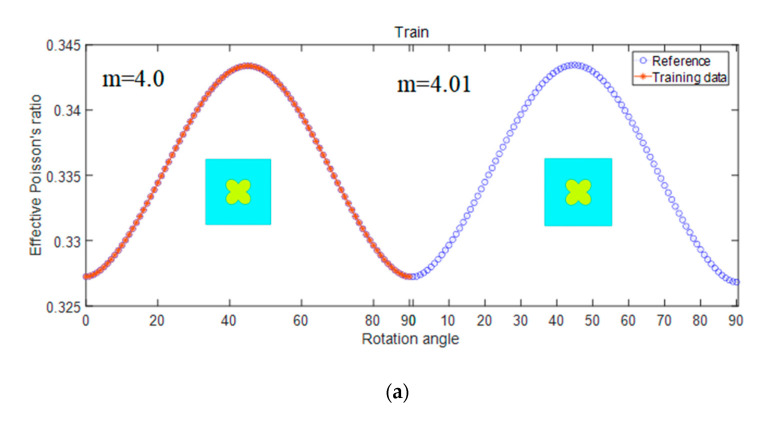

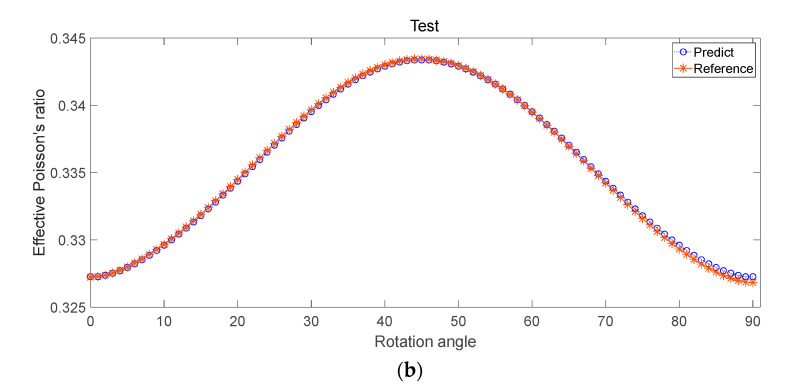
Influence of graph parameters m and rotation angle on effective Poisson’s ratio (**a**) proportion of training set (**b**) prediction.

Subsequently, the investigation focuses on the effective Poisson’s ratio to explore the effects of two parameters: rotation angle and “m”. To showcase the predictive ability of the model for different geometric parameters, we select “m” = 4, “n” = 2, “pr” = 0.88, “$A_{0}$” = 8.712, and the lattice constant is “a” = 10 mm. The influence of geometric parameters “m” and rotation angle on effective Poisson’s ratio is presented in Figure 8, with the specific shapes illustrated in Figure 8a. The results of the predictions are exhibited in Figure 8b. Due to the minimal change in structure, the difference in effective Poisson’s ratio between “m” = 4 and “m” = 4.01 is slight. Therefore, the combination of parameter “m” and rotation angle has a minimal influence on the effective Poisson’s ratio.

### 4.2. The Effective Properties of 3D Fluid-Solid Metamaterials

After discussing the effective properties of the porous material and the solid-solid-based two-dimensional structures of the metamaterials, the effective properties of three-dimensional structures in the case of fluid-solid-based metamaterials are considered. The matrix is set as water, and the thermal conductivity of the scatter is 10 (W/mK). Two classical problems are addressed: hydrostatics and hydrodynamics.

**Figure 9:**
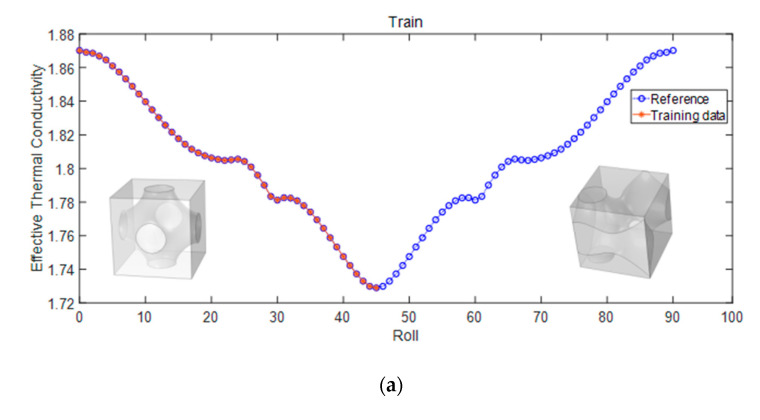

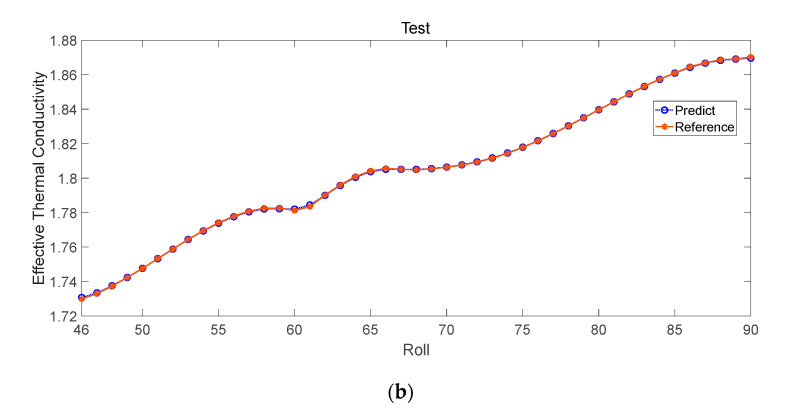
Influence of Roll on effective thermal conductivity (**a**) proportion of training set (**b**) prediction.

**Figure 10:**
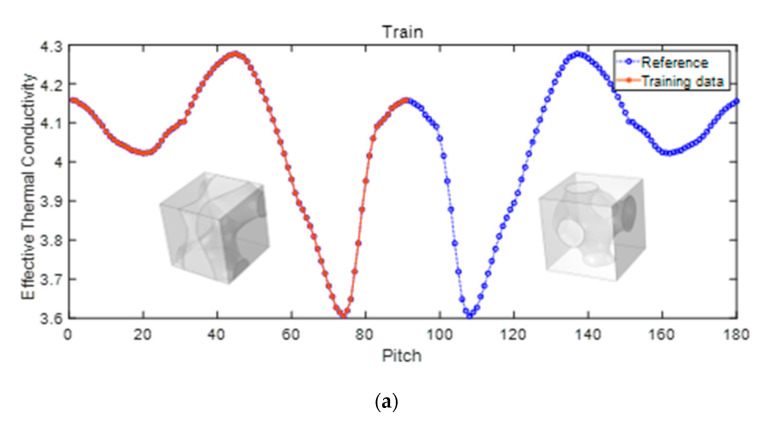

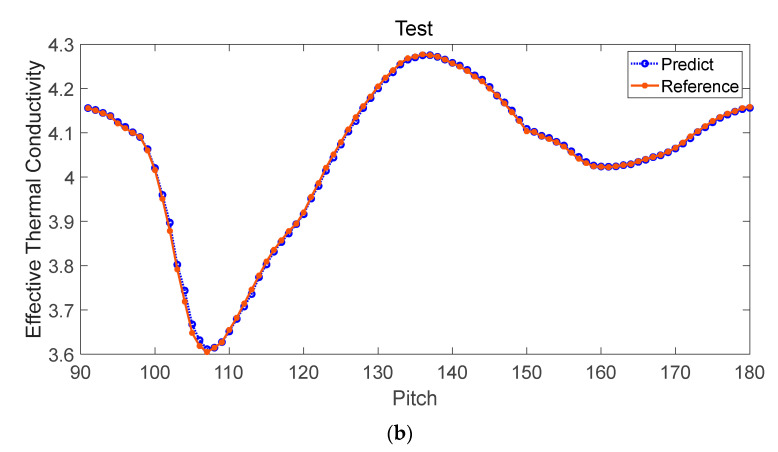
Influence of Pitch on effective thermal conductivity (**a**) proportion of training set (**b**) prediction.

The effective thermal conductivity of the 3D TPMS structure shown in Figure 4c is investigated in this section. Two matrices mentioned in Section 3, Roll and Pitch, are utilized to transform the structure. The influence of these two parameters on the effective thermal conductivity is studied, as shown in Figure 9a and Figure 10a. The parameter Roll is used in the case of the static problem, and the split ratio is set as 50%. The effective thermal conductivity decreases slowly as the water is static. It is symmetrical before and after 45° due to the scatter rotated around the Y-axis when the calculation direction is x-direction. It is evident from Figure 9b that the RF model predicts well.

The influence of the design parameter “Pitch” is quite distinct. The effective thermal conductivity changes from 3.6 to 4.3, which ranges larger compared with the static problem. The alteration of the effective thermal conductivity demonstrates a strong nonlinear behavior, but notably, it forms a symmetrical trend just before and after 90°. This is because the parameter “Pitch” makes a scatter that is rotated around the X-axis when the calculation direction is x-direction. It obtained the same value at 0°, 90° and 180° but the center of symmetry is 90°. The split ratio, as depicted in Figure 10a, remains consistently at 50%. The prediction results obtained from the RF model are shown in Figure 10b. The model satisfactorily predicts both the value and trend of 3D effective thermal conductivity.

### 4.3. Performance of RF

To quantitatively evaluate the precision of the RF model in characterizing metamaterial properties, we utilize metrics including RMSE, MAPE, and $R^{2}$. Smaller RMSE and MAPE values signify superior model performance, while a higher $R^{2}$ value indicates predictions closer to the true values. As illustrated in Table 1, the RF model exhibits high accuracy in its predictions.

### 4.4. Inverse Design of Metamaterials

**Figure 11:**
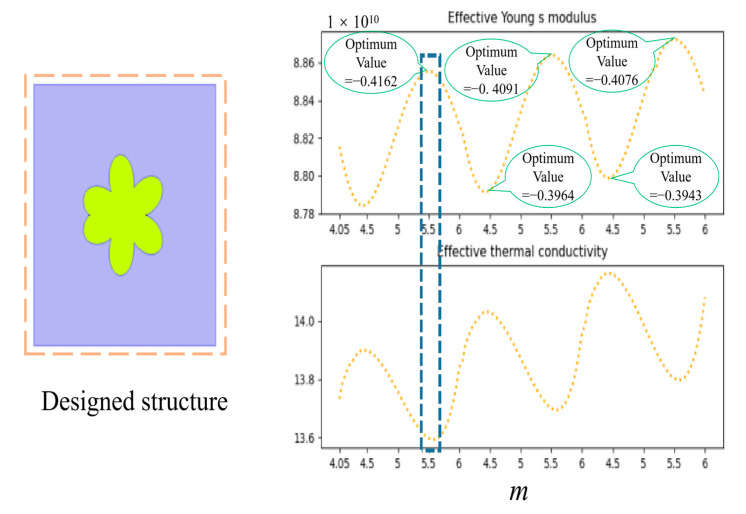
The representative topology construction of the designed metamaterial and the optimal properties point.

In the domain of metamaterial design, a pivotal goal is to craft structures with diverse and outstanding properties. For instance, certain industrial applications necessitate metamaterials characterized by low thermal conductivity and high Young’s modulus, presenting challenges in material selection and design. The optimal results for inverse design in this paper are illustrated in Figure 11. At the optimal solution node, the model strategically selects the best solution, achieving the lowest thermal conductivity when Young’s modulus is relatively large. In this scenario, setting $w_{1}$ higher than 0.5 and $w_{2}$ lower than 0.5 is considered optimal. In this study, the weight for Young’s modulus ($w_{1}$) is set at −0.7, and for equivalent thermal conductivity ($w_{2}$), it is set at 0.3. The AO algorithm performs well, with Young’s modulus reaching an extreme point at “m” = 5.5449 and “ss” = 1.0185, albeit not the highest point. Simultaneously, the thermal conductivity reaches the global lowest point. This discrepancy arises because Young’s modulus does not exhibit significant variations in the subsequent peaks, while the thermal conductivity shows substantial fluctuations. The optimization results are deemed reasonable and suitable in this specific context.

**Figure 12:**
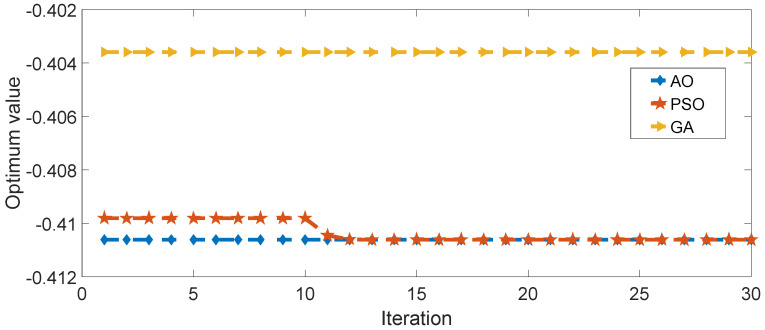
Evolution process of optimal objective function.

To underscore the exceptional performance of the AO algorithm, we conduct a comparison with two alternative algorithms: Particle Swarm Optimizer (PSO) [26] and Genetic Algorithm (GA) [27]. In the case of Particle Swarm Optimizer (PSO), it employs a global-best concept to identify the best solution, and this is refined in each iteration. On the other hand, the Genetic Algorithm (GA), inspired by nature and connected to the principles of Darwin’s theory, mimics the process of biological evolution in its search for optimal solutions. The algorithms undergo 30 iterations with a population size of 40. As illustrated in Figure 12, AO rapidly identifies a viable solution right from the beginning, while PSO is still in the process of searching for an improved one. The objective function value of GA exhibits a noticeable gap compared to the other two algorithms. Table 2 provides a detailed comparison of the optimum value, optimal solution, and calculation time for the three algorithms. AO consumes a relatively lower amount of calculation time than PSO.

## 5. Conclusions

In this study, we propose a machine-learning-based scheme for predicting effective metamaterial properties and inverse designing structures. The corresponding results of this study can be concluded as follows:

(1) For 2D microstructures, the RF regression model predicts the effective Young’s modulus, effective Poisson’s ratio, and effective thermal conductivity of porous and solid–solid metamaterials. The root mean square error, coefficient of determination, and mean absolute percentage error are 0.0002, 0.99945, and 0.0380, respectively.
(2) For 3D microstructures, the RF regression model predicts the effective thermal conductivity of fluid-solid metamaterials in the case of hydrostatics and hydrodynamics. The root mean square error, coefficient of determination, and mean absolute percentage error are 0.0004, 0.99992, and 0.0182, respectively.
(3) Moreover, our study demonstrates that the Aquila Optimizer (AO) performs effectively in solving multi-objective optimization problems. One microstructure with high Young’s modulus and low thermal conductivity is designed.

This research contributes to expediting the design of metamaterials tailored to multiple requirements, addressing the urgent demand for metamaterials with diverse excellent properties in current engineering. However, the shortcoming of this model is that the design space is limited in a parameter domain. It is only valid in the parameter domain of this paper. A no-boundary constraint design will be studied in our next paper.

## Figures and Tables

**Table 1:** RMSE, MAPE, and $R^{2}$ of RF.

Sample	RMSE	MAPE	R^2^	Design Parameters	Dimension
1	0.0354	0.0886	0.99924	Rotation angle	2D
2	0.0008	0.0400	0.98978	Rotation angle	2D
3	0.1190	0.0851	0.98097	m, ss	2D
4	0.0002	0.0380	0.99945	m, rotation angle	2D
5	0.0004	0.0182	0.99992	Roll	3D
6	0.0034	0.0700	0.99907	Pitch	3D

**Table 2:** Performance comparison of three different algorithms.

Algorithm	Optimum Value	Optimal Solution	Time
AO	−0.41062	5.5449 1.0185	50.78
PSO	−0.41061	5.5306 1.0126	69.53
GA	−0.40359	4.0500 1.0000	35.68

## Data Availability

The raw data supporting the conclusions of this article will be made available by the authors on request.
